# Structural Study of the Compounds Formed in the Reactions of FeCl_3_·6H_2_O with Ni(OH)_2_ in the Presence of Dithiolenes HSRSH (R = C_6_H_2_Cl_2_ or C_6_H_4_)

**DOI:** 10.3390/molecules25092240

**Published:** 2020-05-10

**Authors:** Esther Delgado, Elisa Hernández, María Pérez, Josefina Perles, Félix Zamora

**Affiliations:** 1 Departamento de Química Inorgánica, Universidad Autónoma de Madrid, 28049 Madrid, Spain; esther.delgado@uam.es (E.D.); elisa.hernandez@uam.es (E.H.); mprzcor@gmail.com (M.P.);; 2 SCXRD Laboratory, SIdI, Universidad Autónoma de Madrid, 28049 Madrid, Spain; josefina.perles@uam.es; 3 Instituto Madrileño de Estudios Avanzados en Nanociencia, 28049 Madrid, Spain

**Keywords:** transition metal dithiolene compounds, ion-pair compounds, iron-nickel coordination compounds, crystal structure analysis, total reflection X-ray fluorescence analyses

## Abstract

In our attempts to prepare coordination polymers by reaction of FeCl_3_·6H_2_O and Ni(OH)_2_ in the presence of dithiolenes HSC_6_H_2_X_2_SH (X = Cl or H), several ion pairs of compounds containing the anionic entity [Ni(SC_6_H_2_X_2_S)_2_]^−^ were obtained instead. It was also found that other species without dithiolene ligands were formed in these reactions, giving rise to different ion pairs and a tetrametallic cluster. The careful isolation of the different types of crystalline solids allowed the characterization of all of the resulting compounds by single crystal X-ray diffraction (SCXRD). In order to establish the amount of nickel and iron present in the crystals, complementary total reflection X-ray fluorescence (TXRF) analyses were performed. The eight different structural types that were obtained are described and compared with related ones found in the literature.

## 1. Introduction

The chemistry of transition metals with dithiolene derivatives is a research field of high interest due to the outstanding physical properties that these compounds can show, as well as their wide structural diversity [1,2,3,4,5,6,7,8,9,10,11,12,13,14,15]. The dithiolenes are known as “non-innocent” ligands since they can form complexes with metals in different oxidation states giving rise to a rich redox chemistry of both the ligand and the metal center [16], leading to the formation of neutral, monoanionic and dianionic dithiolene derivatives [17]. We have previously studied the influence of several parameters, such as the nature of the dithiolene ligands, the size of the counter cations, the influence of crystallization conditions, as well as the type of iron precursor used, in the synthesis of a series of coordination polymers (CPs) containing the dianionic entities [Fe_2_(SC_6_H_2_X_2_S)_4_]^2−^ (X = Cl or H) and [M_2_(µ-L)_n_]^2+^ (M = alkali metal) as counter-cations [18,19]. More recently, we also described the syntheses of some ion-pair molecules instead of CPs, when divalent cations such as Ca(II), Ba(II), and Zn(II) were used [20].

Taking into account these results, we now evaluated whether the Ni(II) cation, that shows high ability to coordinate to sulfur atoms, could act as a linker between the anionic entities [Fe_2_(SC_6_H_2_X_2_S)_4_]^2−^ (X = Cl or H) in order to prepare CPs. Herein, we report on the syntheses and crystal structures of the new compounds [Ni(DMF)_6_][Ni(SC_6_H_2_Cl_2_S)_2_]_2_·2DMF (**1**), [Ni(DMF)_6_][Fe_2_(µ-O)Cl_6_] (**2**), [Ni_2_(µ-Cl)_3_(THF)_6_][FeCl_4_] (**3**), [Ni_2_(µ-Cl)_3_(THF)_6_][Ni(SC_6_H_2_Cl_2_S)_2_] (**4**), [Ni(DMF)_6_][Ni(SC_6_H_4_S)_2_]_2_·2DMF (**5**), [Ni(DMF)_5_Cl][Ni(SC_6_H_4_S)_2_] (**6**) and [Fe_2.6_Ni_1.4_(µ-Cl)_8_(THF)_6_] (**7**) formed in the reactions between FeCl_3_·6H_2_O with Ni(OH)_2_ in the presence of dithiolenes HSRSH (R = C_6_H_2_Cl_2_ or C_6_H_4_). It has to be noticed that models obtained from single-crystal X-ray diffraction (SCXRD) data of some heterobimetallic structures cannot properly differentiate between iron and nickel atoms, two metal centers with very similar electron densities. Then, a total reflection X-ray fluorescence (TXRF) analysis on the measured single crystals was successfully applied to calculate the element content and to refine the occupation of the metal positions [21].

## 2. Results and Discussion

Previously, we have observed that the method used for the syntheses of CPs with [Fe_2_(SC_6_H_2_Cl_2_S)_4_]^2−^ and different alkali metals [20] failed in our attempts to prepare CPs with the same dianionic entity and divalent metals such as Ca(II), Ba(II) and Zn(II) as counter-cations.

**Scheme 1:**
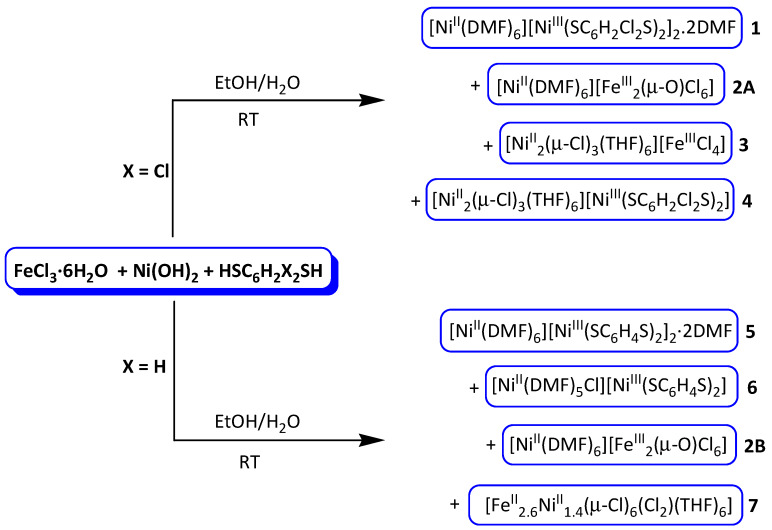
Schematic representation of the reactions carried out to isolate compounds **1**–**7**.

Taking into account these results, we decided to evaluate the role of Ni^2+^ in the reaction with FeCl_3_·6H_2_O and HSC_6_H_2_Cl_2_SH, using Ni(OH)_2_ as a deprotonating agent (Scheme 1). Thus, after stirring a mixture of FeCl_3_·6H_2_O, HSC_6_H_2_Cl_2_SH and Ni(OH)_2_ for 24 h at room temperature, the precipitate obtained was crystallized in DMF/Diethyl ether (1:4) giving rise to green crystals of [Ni(DMF)_6_][Ni(SC_6_H_2_Cl_2_S)_2_]_2_·2DMF (**1**) together with yellow crystals of [Ni(DMF)_6_][Fe_2_(µ-O)Cl_6_] (**2A**), as major and minor compounds of the reaction, respectively. Furthermore, after removing in vacuum the solvent of the initial filtrate and dissolving the solid residue in THF/*n*-heptane, a mixture of crystals of compounds [Ni_2_(µ-Cl)_3_(THF)_6_][FeCl_4_] (**3**) and [Ni_2_(µ-Cl_3_)(THF)_6_][Ni(SC_6_H_2_Cl_2_S)_2_] (**4**) were obtained in trace amounts.

On the other hand, it is well known that the nature of the dithiolene ligands can affect the formation of different metal-dithiolene derivatives. Taking into account this fact, we considered it interesting to carry out a similar reaction as described above using HSC_6_H_4_SH instead in order to compare the behavior of both dithiolenes. Thus, the solid compound obtained after stirring the mixture of FeCl_3_·6H_2_O, HSC_6_H_4_SH and Ni(OH)_2_ for 24 h, was crystallized in DMF/Diethyl ether, yielding [Ni(DMF)_6_][Ni(SC_6_H_4_S)_2_]_2_·2DMF (**5**) as the main reaction product, together with trace amounts of compounds [Ni(DMF)_5_Cl][Ni(SC_6_H_4_S)_2_]_2_ (**6**) and [Ni(DMF)_6_][Fe_2_(µ-O)Cl_6_] (**2B**) (Scheme 1). Additionally, the residue obtained after removing the solvent of the filtrate in the reaction was recrystallized from a THF/*n*-heptane mixture, yielding a few yellow crystals of [Fe_2.6_Ni_1.4_(µ-Cl)_8_(THF)_6_] (**7**).

All of the different compounds were isolated and analyzed by single-crystal X-ray diffraction (SCXRD), and their crystal structures were solved. The existence of both Ni and Fe atoms in the reaction media represented a problem for the structural determination, as both metals showed similar electron densities, making it very difficult to assign the occupation of the metal centers in the electron density maps solely based on the data obtained by SCRXD. To overcome this problem, TXRF analysis (Supplementary Materials section S2) was performed on the selected and measured single crystals, in order to state the chemical nature of the metals present in the crystalline solids (Table S27).

**Figure 1:**
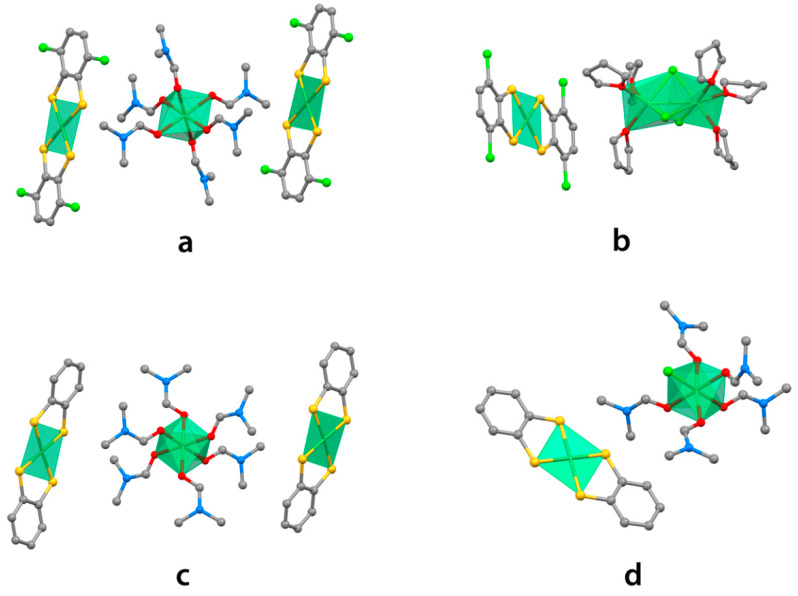
Ionic species per formula unit found in the dithiolene derivatives: **a**, [Ni(DMF)_6_][Ni(SC_6_H_2_Cl_2_S)_2_]_2_ **1**; **b**, [Ni_2_(µ-Cl_3_)(THF)_6_][Ni(SC_6_H_2_Cl_2_S)_2_] **4**; **c**, [Ni(DMF)_6_][Ni(SC_6_H_4_S)_2_]_2_ **5**; and **d**, [Ni(DMF)_5_Cl][Ni(SC_6_H_4_S)_2_] **6**. Green octahedra and square planar geometries represent Ni coordination environments. Oxygen atoms are depicted in red, sulfur ones in yellow, nitrogen in blue, chlorine in light green, and carbon in grey. Solvent molecules and hydrogen atoms have been omitted for clarity.

As depicted in Figure 1, the resulting compounds **1** and **4**-**6** contained the anionic entity [Ni(SC_6_H_2_X_2_S)]^−^ (X = Cl, H) in their structure, where Ni adopts the commonly found square planar coordination environment NiS_4_ with Ni-S (2.136(2)–2.1570(9) Å) distances and angles (Tables S17 and S18 in the Supplementary Materials) similar to those found in other related compounds [22,23,24]. In these four structures, the negative charge is balanced with cationic species derived from the coordination of the solvent molecules to another nickel atom.

Compound **1** (Figure 1a) crystallizes as green prisms in the *P*2_1_/*n* space group with half a [Ni(DMF)_6_]^2+^ cation, one [Ni(SC_6_H_2_Cl_2_S)_2_]^−^ anion, and one DMF molecule in the asymmetric unit, yielding a general formula [Ni(DMF)_6_][Ni(SC_6_H_2_Cl_2_S)_2_]_2_·2DMF. Compound **5** (Figure 1c) obtained in the reaction with HSC_6_H_4_SH instead of the chlorinated one, presented an analogous formula {[Ni(DMF)_6_][Ni(SC_6_H_4_S)_2_]_2_·2 DMF}. It crystallized as small green plates in the *P*2_1_/*c* space group, also with half a [Ni(DMF)_6_]^2+^ cation, one [Ni(SC_6_H_2_Cl_2_S)_2_]^−^ anion, and one DMF molecule in the asymmetric unit. In both cases, the nickel atom in the cationic moiety is coordinated to six oxygen atoms from six DMF ligands in an octahedral NiO_6_ geometry, while in the anionic species, Ni^2+^ is coordinated to four sulfur atoms from two dithiolene ligands, as explained above. Both Ni-O (2.035(3)-2.065(4) Å) [25,26,27] distances and angles (Tables S17 and S18) are similar to those found in other related compounds. The TXRF measurements for compounds **1** and **5** confirm that only nickel atoms are present in both metal positions (Tables S20 and S24).

Compound **4**, with formula [Ni_2_(µ-Cl_3_)(THF)_6_][Ni(SC_6_H_2_Cl_2_S)_2_] (Figure 1b), crystallized as green prisms in the *P*-1 space group with a [Ni_2_(µ-Cl_3_)(THF)_6_]^+^ cation, and two halves of the mentioned [Ni(SC_6_H_2_Cl_2_S)_2_]^−^ anionic molecules. In the cationic moiety, the Ni atom is coordinated to three oxygen atoms from the THF molecules and three bridge chlorine atoms, and the two NiO_3_Cl_3_ octahedra are sharing the triangular Cl-Cl-Cl face. This cation has been found in the literature in compounds [Ni_2_(µ-Cl_3_)(THF)_6_][SnCl(THF)_6_] [28], and [Ni_2_(µ-Cl_3_)(THF)_6_][NMe_4_][NiSn_4_C_5_H_12_Cl_8_O] [29], and the observed Ni-Ni (2.9849(5) Å), Ni-O (2.059(2)-2.115(2) Å) and Ni-Cl (2.4002(9)-2.4299(9) Å) distances and angles in **4** (Tables S17 and S18) agree with the reported values. The TXRF analysis (Table S23) for compound **4** shows a small content of iron (approximately 12%) apparently due to the deposition on the surface of the crystals of some iron-containing compounds, present in the reaction media, as amorphous or nanocrystalline solids.

Compound **6** (Figure 1d) crystallized as green prisms in the *P*-1 space group with a [Ni (DMF)_5_Cl]^+^ cation and two halves of the common [Ni(SC_6_H_2_S)_2_]^−^ anionic molecules, for a sum formula [Ni(DMF)_5_Cl][Ni(SC_6_H_4_S)_2_]. The [Ni(SC_6_H_2_S)_2_]^−^ anion presents the expected square planar NiS_4_ geometry, while the nickel atom in the cation is surrounded by five oxygen atoms from five DMF molecules (Ni-O distances in the range 2.065(2)–2.107(2) Å, see Table S17) plus a chlorine atom (Ni-Cl distance of 2.3723(8) Å, Table S17) in a distorted octahedral NiO_5_Cl environment, similar to the one described in the bibliography for this moiety in [Ni(DMF)_5_Cl][Ni(Se_2_C_2_(CN)_2_)_2_] [30].

**Figure 2:**
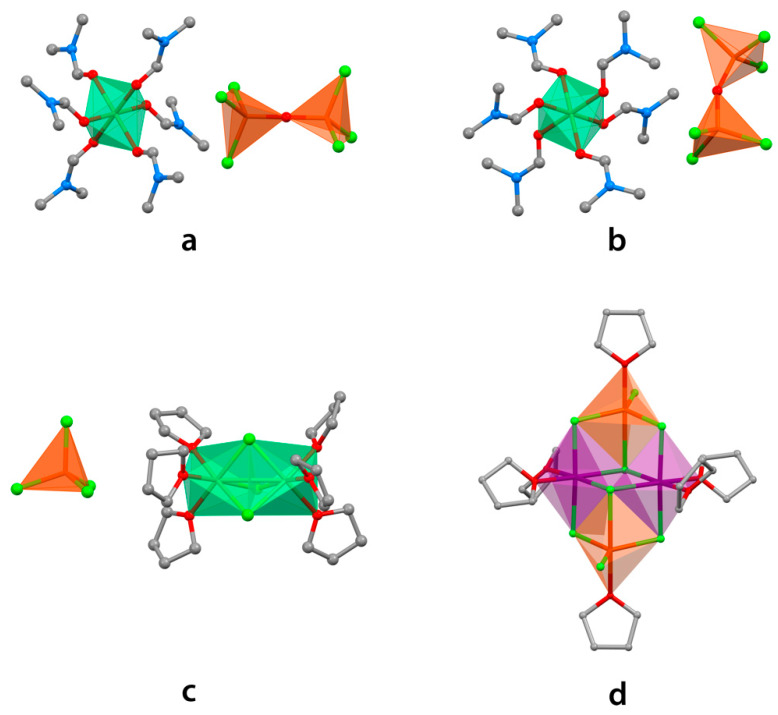
Molecular species per formula unit in the compounds containing both Fe and Ni metals: **a**, [Ni(DMF)_6_][Fe_2_(µ-O)Cl_6_] **2A**; **b**, [Ni(DMF)_6_][Fe_2_(µ-O)Cl_6_] **2B**; **c**, [Ni_2_(µ-Cl)_3_(THF)_6_][FeCl_4_] **3**; **d**, tetrametallic compound [M_4_(µ-Cl)_8_(THF)_6_] **7**; hydrogen atoms have been omitted for clarity. Green octahedra represent Ni coordination environments; orange tetrahedra, Fe environments; and purple octahedra the shared Fe/Ni ones; oxygen atoms are depicted in red, nitrogen in blue, chlorine in light green and carbon in grey.

Additionally, compounds **2A**, **2B**, and **3** (Figure 2a–c) were also obtained and isolated in the reactions described in the experimental section (Section 3.1.1 and Section 3.1.2). These three compounds contained nickel cationic moieties that were already present in compounds **1** and **5** ([Ni(DMF)_6_]^2+^) and **4** ([Ni_2_(µ-Cl_3_)(THF)_6_]^+^), together with some iron anions derived from the FeCl_3_·6H_2_O reactant.

Compound **2A**, with formula [Ni(DMF)_6_][Fe_2_(µ-O)Cl_6_] (Figure 2a), crystallized as yellow prisms and was found to be isostructural with a previously reported manganese-iron compound with formula [Mn(DMF)_6_][Fe_2_(µ-O)Cl_6_] [31], which had been isolated in a reaction to obtain heterometallic complexes using FeCl_3_·6H_2_O as reactant. It includes the cationic entity [Ni(DMF)_6_]^2+^ (found in compounds **1** and **5**) and the anionic dimer [Fe_2_(µ-O)(Cl)_6_]^2−^. This anion is composed of two fused FeCl_3_O tetrahedra sharing the central oxygen vertex. The Fe-Cl and Fe-O distances are 2.231(9) Å and 1.7663(9) Å, respectively, in the range expected for this species. The Fe-O-Fe angle has a value of 180^°^, and the two iron tetrahedra adopt a staggered conformation as a consequence of the O and Fe locations at positions with -3 and 3 symmetry, respectively. The formation of the [Fe_2_(µ-O)(Cl)_6_]^2−^ anion in this reaction may be the result of the partial hydrolysis of either FeCl_3_ or [FeCl_4_]^−^ by oxygen from water [32]. TXRF measurements in compound **2A** (Table S21) confirmed the presence in the crystal of both nickel and iron atoms in a 1:2 ratio. Compound **2B** (Figure 2b) was found to be a new structural type, a polymorph of compound **2A**, with a slightly different geometry for the [Fe_2_(µ-O)(Cl)_6_]^2−^ anion. Fe-Cl (2.222(1)-2.219(2) Å) and Fe-O (1.753(3) Å) distances are slightly shorter than the ones found in **2A**. In this case, the Fe-O-Fe angle in the anion is 154.2(2)°, closer to the most frequent values found in the literature for this molecule [33,34], and resulting in a different and less dense supramolecular arrangement (Figure S4).

Compound [Ni_2_(µ-Cl_3_)(THF)_6_][FeCl_4_] **3** (Figure 2c) appeared as yellow prismatic polyhedra and crystallized in the monoclinic *P*2_1_/*c* space group. A structural search in the Cambridge Structural Database found that **3** is isostructural with reported compounds [Mg_2_(µ-Cl_3_)(THF)_6_][FeCl_4_] [35] and [V_2_(µ-Cl_3_)(THF)_6_][FeCl_4_] [36]. In the dinuclear cation [Ni_2_(µ-Cl_3_)(THF)_6_]^+^, analogous to the one found in compound **4**, each Ni atom is coordinated to three THF molecules (Ni-O distances from 2.070(4) to 2.104(4) Å) and three bridging chloride ligands (Ni-Cl 1.395(2)–1.439(2) Å), yielding distorted NiO_3_Cl_3_ octahedra, and the two polyhedra share the Cl-Cl-Cl face. Regarding the [FeCl_4_]^−^ anion, the usual tetrahedral geometry was observed around the iron atom (Fe-Cl 1.156(2)–1.184(2) Å) as a consequence of the coordination of four chloride ligands to this metal. The TXRF measurements carried out on the crystal of **3** (Table S22) confirmed the presence in the molecule of nickel and iron atoms in the expected 2:1 ratio.

Compound **7** (Figure 2d) crystallized in the triclinic *P*-1 space group and was isostructural with the iron [Fe_4_(µ-Cl)_8_(THF)_6_] [37,38] and the cobalt [Co_4_(µ-Cl)_8_(THF)_6_] [39] derivatives. As described in the previous reports [37,38,39], it consists of a tetrametallic cluster of edge-sharing polyhedra related to the inorganic CdI_2_ structure, with two metal positions in the asymmetric unit (M_A_ and M_B_). The M_A_ location displays a M_A_Cl_4_O trigonal bipyramidal environment (M_A_-Cl between 2.252(1) and 2.7018(8) Å and M_A_-O 2.130 (2) Å) while the M_B_ site displays an M_B_Cl_4_O_2_ octahedral geometry with the two oxygen atoms in a *cis* disposition (M_B_-Cl between 2.4375(8) and 2.4814(8) Å) and M_B_-O 2.087(2), 2.115(2) Å). As the TXRF measurements carried out on the crystal of compound **7** (Figure S9) confirmed the presence in the structure of Ni:Fe atoms in a 0.35:0.65 ratio, the model was refined with a 100% occupation of position M_A_ by iron atoms (with label Fe1) and the M_B_ position shared by iron and nickel atoms in a 70% Ni:30% Fe ratio (with atoms labeled Ni1/Fe2). This assignation was based on the fact that compounds with Fe atoms displaying both metal environments were found in the literature; however, for Ni atoms, no structures with M_A_Cl_4_O trigonal bipyramidal geometry were reported, but two structures displaying analogous MCl_4_O_2_ octahedral geometry (with two *cis* oxygen atoms) could be found [40,41].

## 3. Materials and Methods

All the reagents and solvents are commercially available and were used as received without further purification. Syntheses of all compounds were carried out under argon atmosphere at room temperature.

Elemental analysis measurements were conducted with a LECO CHNS-932 (Model NO: 601-800-500) microelemental analyser (LECO, St. Joseph, Mich., USA). TXRF (Total reflection X-Ray Fluorescence) analysis of the samples was performed with a benchtop S2 PicoFox TXRF spectrometer (Bruker Nano, Berlin, Germany), equipped with a molybdenum X-ray source working at 50 kV, 600 µA and 500 s and an XFlash SDD detector (effective area of 30 mm^2^) and an energy resolution better than 150 eV for Mn K_α_. The Spectra 7 software package, also from Bruker, was used for control, acquisition, deconvolution, and integration of all analyzed samples.

Single-crystal X-ray diffraction (SCXRD) measurements were collected in a Bruker Kappa Apex II diffractometer using graphite-monochromated Mo K_α_ radiation (λ = 0.71073 Å) Single crystals of each of the compounds were isolated and covered with a layer of an inert mineral oil, mounted on a MiTeGen micromount with the aid of a microscope (MiTeGen, Ithaca, NY), and immediately placed in the low temperature nitrogen stream at 200 K from an Oxford Cryostream (Oxford Cryosystems, Oxford, United Kingdom) 700 unit. A summary of selected crystal and refinement data can be found in Table 1 and Table 2 and more comprehensive information is collected in the Supplementary Material (Tables S1–S16). Representations of the labelled asymmetric units for each one of the crystal structures are collected in Figures S1 (**1**), S2 (**2A**), S3 (**2B**), S5 (**3**), S6 (**4**), S7 (**5**), S8 (**6**) and S9 (**7**). Metal environment parameters are listed in Tables S17 (distances) and S18 (angles), and the existent weak C-H···S, C-H···O, and C-H···Cl supramolecular interactions are gathered in Table S19. CCDC 1834782 (**1**), 1989776 (**2A**), 1989767 (**2B**), 1989768 (**3**), 1989769 (**4**), 1989770 (**5**), 1989771 (**6**), and 1989772 (**7**) contain the crystallographic data for the crystal structures. Structural data can be obtained free of charge from the Cambridge Crystallographic Data Centre via www.ccdc.cam.ac.uk/data_request/cif.

### 3.1. Synthesis

#### 3.1.1. Reaction of FeCl_3_·6H_2_O and Ni(OH)_2_ in the presence of HSC_6_H_2_Cl_2_SH

A mixture of HSC_6_H_2_Cl_2_SH (162 mg, 0.78 mmol) and Ni(OH)_2_ (93 mg, 1 mmol) in 10 mL of EtOH-H_2_O (1:1) was stirred at room temperature for 2 h, then a solution of FeCl_3_·6H_2_O (102 mg, 0.38 mmol) in 10 mL of EtOH-H_2_O (1:1) was added. The mixture was stirred for 24 h and then filtered off. The solid obtained in this reaction was crystallized at −20 °C in DMF/diethyl ether (1:4) giving rise to green crystals of [Ni^II^(DMF)_6_][Ni^III^SC_6_H_2_Cl_2_S)_2_]_2_·2DMF, (**1**) (52 mg, 34.3% yield) together with yellow crystals of compound [Ni^II^(DMF)_6_][Fe^III^_2_(µ-O)Cl_6_], (**2A**) (29 mg, 9% yield). Anal. Calcd. (Found) for C_48_H_64_Cl_8_N_8_Ni_3_O_8_S_8_ (**1**): C, 36.16 (36.08); H, 4.05 (4.05); N, 7.03 (6.88); S, 16.09 (15.70). Anal. Calcd. (Found) for C_21_H_49_Cl_6_Fe_2_N_7_NiO_8_ (**2A**+1DMF): C, 27.69 (28.22); H, 5.42 (5.75); N, 10.77 (9.74).

The residue obtained after removing the solvent was extracted with 5 mL of THF, and further crystallization at room temperature in THF/*n*-heptane (1:1) yielded a mixture of crystals consisting of: yellow prisms [Ni^II^_2_(µ-Cl)_3_(THF)_6_][Fe^III^Cl_4_], (**3**), and green ones of [Ni^II^_2_(µ-Cl_3_)(THF)_6_][Ni^III^(SC_6_H_2_Cl_2_S)_2_], (**4**), both in trace amounts.

#### 3.1.2. Reaction of FeCl_3_·6H_2_O and Ni(OH)_2_ in the presence of HSC_6_H_4_SH

A similar procedure followed in the reaction described above was carried out, but using HSC_6_H_4_SH, instead. The residue obtained from the reaction was crystallized at −20 °C in DMF/diethyl ether (1:4) giving rise to green crystals of [Ni^II^(DMF)_6_][Ni^III^(SC_6_H_4_S)_2_]_2_·2DMF (**5**) as the main product (234 mg, 26% yield), together with some other green and yellow crystals of [Ni^II^(DMF)_5_Cl][Ni^III^(SC_6_H_4_S)_2_] (**6**) and [Ni^II^(DMF)_6_][Fe^III^_2_(µ-O)Cl_6_] (**2B**) respectively, as trace amounts.

Anal. Calcd. (Found) for C_45_H_65_N_7_Ni_3_O_7_S_8_ (**5** -1 DMF): C, 43.29 (44.12); H, 5.25 (5.23); N, 7.85 (6.74); S, 20.54 (20.67).

Additionally, the residue obtained by removing the solvent in the initial filtrate was crystallized at room temperature in THF/*n*-heptane (1:1), giving rise to few yellow crystals of compound [Fe^II^_2.6_Ni^II^_1.4_(µ-Cl)_8_(THF)_6_] (**7**).

Please note that in some cases, the experimental analyses were not fully satisfactory. This is due to the moderate-to-low stability of the coordination compounds, showing volatile molecules of solvent weakly coordinated to the metal center. This behavior has been previously reported for related compounds [16,18,19,20].

## 4. Conclusions

Homo- or hetero-bimetallic ion-pairs were obtained, instead of coordination polymers, from the reactions between FeCl_3_·6H_2_O, Ni(OH)_2,_ and the dithiolates HSC_6_H_2_X_2_SH (X = Cl or H). The analysis of the products formed in these reactions clearly shows the higher tendency of nickel versus iron to coordinate to sulphur atoms, giving rise to the formation of [Ni_2_(SC_6_H_2_X _2_S)_4_]^2−^ (X = Cl or H) instead of [Fe_2_(SC_6_H_2_X _2_S)_4_]^2−^ (X = Cl or H). Eight different crystalline phases (**1**, **2A**, **2B**, **3**, **4**, **5**, **6**, and **7**) were obtained from these reactions and characterized by SCXRD and other techniques.

TXRF analyses were used for the identification and quantification of the metal atoms present in the solids. This technique was particularly useful, as it could be applied to very small quantities of sample and could be performed on the crystals measured in SCXRD. The analyses of the different types of solids allowed us to better understand the species that are formed, both during the reactions and in the crystallization processes.

## Figures and Tables

**Table 1:** Crystal and refinement data for compounds **1**, **2A**, **2B**, and **3**.

	1	2A	2B	3
Formula	C_48_H_64_Cl_8_N_8_Ni_3_O_8_S_8_	C_9_H_21_Cl_3_FeN_3_Ni_0.50_O_3.50_	C_18_H_42_Cl_6_Fe_2_N_6_NiO_7_	C_12_H_4_Cl_4_FeNiS_4_
Space group	*P*2_1_/*n*	*R*-3	*P*-1	*P*2_1_/*c*
*a*/Å	8.730(8)	14.193(2)	9.1594(3)	12.999(1)
*b*/Å	17.144(6)	14.193(2)	9.3113(3)	12.1054(8)
*c*/Å	23.789(8)	15.147(3)	22.1594(8)	23.509(2)
*α*/°	90	90	82.911(2)	90
*β*/°	101.06(4)	90	80.829(2)	90.899
*ɣ*/°	90	120	87.782(2)	90
*V*/Å^3^	3500(4)	2642.4(9)	1851.1(1)	3698.7(5)
*Z*	2	6	2	4
*d* calc/*g*·cm^−^^3^	1.515	1.579	1.503	1.534
*μ*/mm^−^^1^	1.394	1.839	1.750	1.933
*R* indices (*I* > 2σ(*I*))	R_1_ = 0.0687 *w*R_2_ = 0.1325	R_1_ = 0.0362 *w*R_2_ = 0.0738	R_1_ = 0.0535 *w*R_2_ = 0.1222	R_1_ = 0.0569 *w*R_2_ = 0.1663
GooF on *F^2^*	1.085	1.006	1.059	1.119

**Table 2:** Crystal and refinement data for compounds **4**, **5**, **6**, and **7**.

	4	5	6	7
Formula	C_36_H_52_Cl_7_FeNi_2_O_6_S_4_	C_48_H_72_N_8_Ni_3_O_8_S_8_	C_27_H_43_ClN_5_Ni_2_O_5_S_4_	C_24_H_48_Cl_8_Fe_2.6_Ni_1.4_O_6_
Space group	*P*-1	*P*2_1_/*c*	*P*-1	*P*-1
*a*/Å	9.7565(3)	8.4611(4)	8.718(1)	9.99511(6)
*b*/Å	14.2853(5)	18.882(1)	12.212(1)	10.4813(5)
*c*/Å	18.0305(6)	19.522(1)	17.889(2)	10.8907(7)
*α*/°	96.762(2)	90	101.414(1)	62.650(2)
*β*/°	90.792(2)	90.197(3)	97.572(4)	67.991(3)
*ɣ*/°	109.047(2)	90	99.593(5)	82.248(3)
*V*/Å^3^	2355.2(1)	3118.9(3)	1813.8(4)	934.4(1)
*Z*	2	2	2	1
*d* calc/*g*·cm^−^^3^	1.598	1.407	1.463	1.677
*μ*/mm^−^^1^	1.802	1.217	1.383	2.290
*R* indices (*I* > 2σ(*I*))	R_1_ = 0.0326 *w*R_2_ = 0.0834	R_1_ = 0.0297 *w*R_2_ = 0.0688	R_1_ = 0.0312 *w*R_2_ = 0.0758	R_1_ = 0.0310 *w*R_2_ = 0.0772
GooF on *F^2^*	1.070	1.086	1.091	1.031

## Supplementary Materials

The following are available online, crystal structure data for compounds **1**–**7**; section S1. Metal coordination environment parameters; section S2. Supramolecular interactions; section S3. TXRF analyses for compounds **1**, **2A**, and **3**–**7**; section S4.
