# Supplementary material for: Structural Study of the Compounds Formed in the Reactions of FeCl3·6H2O with Ni(OH)2 in the Presence of Dithiolenes HSRSH (R = C6H2Cl2 or C6H4)
Source: Molecules. 2020 May 10;25(9):2240. doi: 10.3390/molecules25092240 (PMC7248742; doi:10.3390/molecules25092240)
Supplement: Supplementary file 1 [file molecules-25-02240-s001.pdf]

Structural study of compounds formed in the reactions of $FeCl_3·6H_2O$ with $Ni(OH)_2$ in the presence of the dithiolenes HSRSH (R= $C_6H_2Cl_2$ or C_6H_4)

Esther Delgado,[1] Elisa Hernández,[1] María Pérez,[1] Josefina Perles[2] and Félix Zamora[1,3,]*

[1] Departamento de Química Inorgánica, Universidad Autónoma de Madrid, 28049 Madrid, Spain
[2] SCXRD Laboratory, SIdI, Universidad Autónoma de Madrid, 28049 Madrid, Spain
[3] Instituto Madrileño de Estudios Avanzados en Nanociencia, 28049 Madrid, Spain;

S1. Crystal structure data for compounds 1-7

1. Model and refinement data

a. Compound 1 (CCDC 1834782)

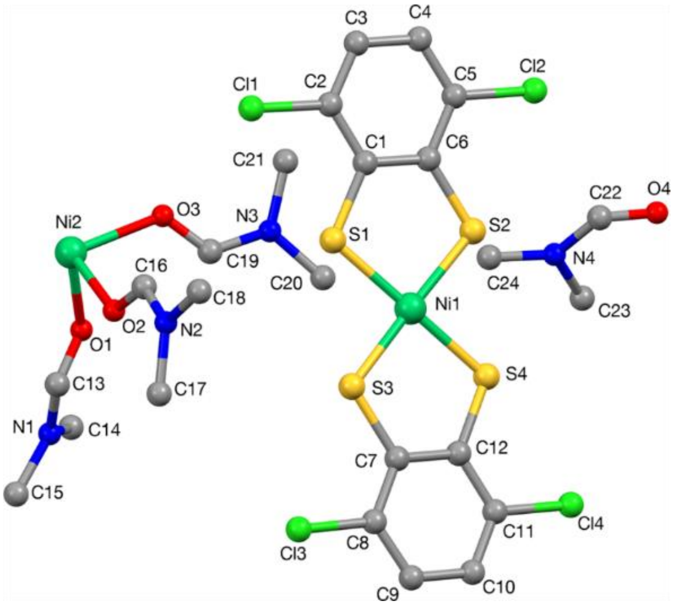

Figure S1. Asymmetric unit of compound **1** with atoms labelled.

Table S1. Sample and crystal data for **1.**

**CCDC Number**	1834782	
**Chemical formula**	$C_{48}H_{64}Cl_8N_8Ni_3O_8S_8$	
**Formula weight**	1597.28 g/mol	
**Temperature**	200(2) K	
**Wavelength**	0.71073 Å	
**Crystal size**	0.120 x 0.270 x 0.280 mm	
**Crystal system**	monoclinic	
**Space group**	$P2_1/n$	
**Unit cell dimensions**	a = 8.730(8) Å	α = 90°
	b = 17.174(6) Å	β = 101.06(4)°
	c = 23.789(8) Å	γ =90°
**Volume**	3500.(4) Å^3	
**Z**	2	
**Density (calculated)**	1.515 g/cm^3	
**Absorption coefficient**	1.394 mm^{-1}	
**F(000)**	1640	

Table S2. Data collection and structure refinement for **1.**

**Theta range for data collection**	3.18 to 27.53°		
**Index ranges**	-11<=h<=11, -22<=k<=22, -30<=l<=29		
**Reflections collected**	15645		
**Independent reflections**	8035 [R(int) = 0.0858]		
**Coverage of independent reflections**	99.6%		
**Absorption correction**	multi-scan		
**Max. and min. transmission**	0.8510 and 0.6960		
**Structure solution technique**	direct methods		
**Structure solution program**	SHELXS-97 (Sheldrick 2008)		
**Refinement method**	Full-matrix least-squares on F^2		
**Refinement program**	SHELXL-2014/7 (Sheldrick, 2014)		
**Data/restraints/parameters**	8035 / 0 / 384		
**Goodness-of -fit on F^2**	1.004		
**Final R indices**	4646 data; I2σ(I)	R_1 = 0.0565	wR_2 = 0.1125
	all data	R_1 = 0.1184	wR_2 = 0.1270
**Weighting scheme**	$w=1/[\sigma 2(F_o^2)+(0.0485P)^2]$ where $P=(F_o^2+2F_c^2)/3$		
**Largest diff. peak and hole**	0.950 and -0.679 eÅ^{-3}		

b. Compound 2A (CCDC 1989776)

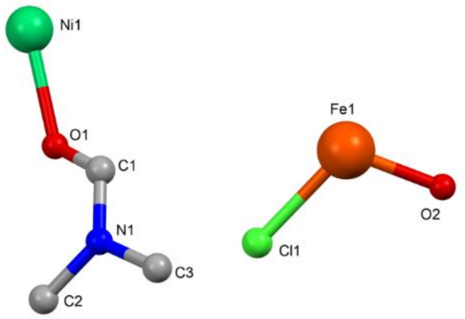

Figure S2. Asymmetric unit of compound **2A** with atoms labelled.

Table S3. Sample and crystal data for **2A.**

**CCDC Number**	1989776	
**Chemical formula**	$C_9H_{21}Cl_3FeN_3Ni_{0.50}O_{3.50}$	
**Formula weight**	418.84 g/mol	
**Temperature**	200(2) K	
**Wavelength**	0.71073 Å	
**Crystal size**	0.030 x 0.040 x 0.070 mm	
**Crystal habit**	intense yellow prismatic	
**Crystal system**	trigonal	
**Space group**	*R* -3	
**Unit cell dimensions**	a = 14.193(2) Å	α = 90°
	b = 14.193(2) Å	β = 90°
	c = 15.147(3) Å	γ = 120°
**Volume**	2642.4(9) $Å^3$	
**Z**	6	
**Density (calculated)**	1.579 g/cm^3	
**Absorption coefficient**	1.839 mm^{-1}	
**F(000)**	1290	

Table S4. Data collection and structure refinement for **2A.**

**Theta range for data collection**	2.13 to 25.33°		
**Index ranges**	-16<=h<=13, -16<=k<=17, -18<=l<=18		
**Reflections collected**	7029		
**Independent reflections**	1080 [R(int) = 0.0743]		
**Coverage of independent reflections**	100.0%		
**Absorption correction**	multi-scan		
**Refinement method**	Full-matrix least-squares on F^2		
**Refinement program**	SHELXL-2014/7 (Sheldrick, 2014)		
**Function minimized**	$\Sigma w(F_o^2 - F_c^2)^2$		
**Data / restraints / parameters**	1080 / 0 / 64		
**Goodness-of-fit on F^2**	1.006		
**Final R indices**	769 data;I>2σ(I)	R_1 = 0.0362	wR_2 = 0.0738
	all data	R_1 = 0.0687	wR_2 = 0.0889
**Weighting scheme**	$w=1/[\sigma^2(F_o^2)+(0.0374P)^2+1.9713P]$ where $P=(F_o^2+2F_c^2)/3$		
**Largest diff. peak and hole**	0.320 and -0.569 $eÅ^{-3}$		

c. Compound 2B (CCDC 1989767)

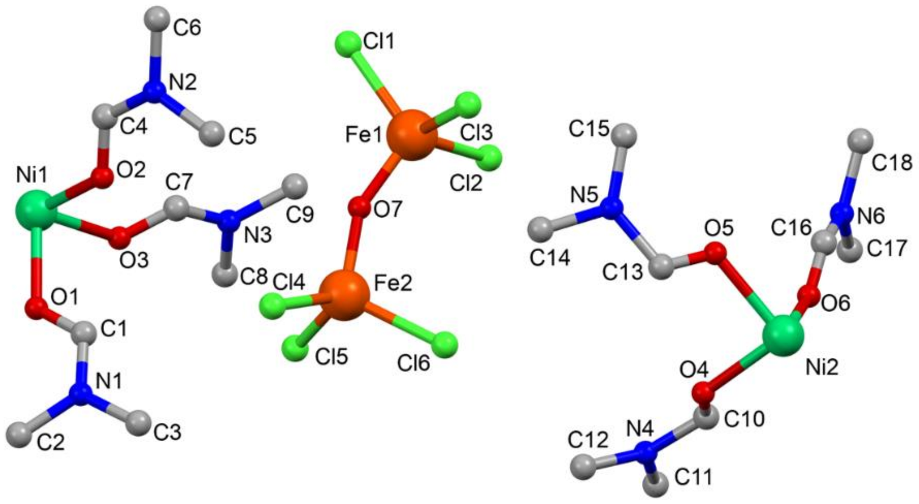

Figure S3. Asymmetric unit of compound **2B** with atoms labelled.

Table S5. Sample and crystal data for **2B.**

**CCDC Number**	1989767	
**Chemical formula**	$C_{18}H_{42}Cl_6Fe_2N_6NiO_7$	
**Formula weight**	837.68 g/mol	
**Temperature**	200(2) K	
**Wavelength**	0.71073 Å	
**Crystal size**	0.040 x 0.090 x 0.240 mm	
**Crystal habit**	clear yellow prismatic	
**Crystal system**	triclinic	
**Space group**	*P* -1	
**Unit cell dimensions**	*a* = 9.1594(3) Å	α = 82.911(2)°
	b = 9.3113(3) Å	β = 80.829(2)°
	c = 22.1594(8) Å	γ = 87.782(2)°
**Volume**	1851.11(11) Å^3	
**Z**	2	
**Density (calculated)**	1.503 g/cm^3	
**Absorption coefficient**	1.750 mm^{-1}	
**F(000)**	860	

Table S6. Data collection and structure refinement for **2B.**

**Theta range for data collection**	1.88 to 25.35°		
**Index ranges**	-10<=h<=11, -11<=k<=11, -26<=l<=26		
**Reflections collected**	49210		
**Independent reflections**	6766 [R(int) = 0.0492]		
**Coverage of independent reflections**	99.9%		
**Absorption correction**	multi-scan		
**Max. and min. transmission**	0.9330 and 0.6790		
**Structure solution technique**	direct methods		
**Structure solution program**	SHELXS-97 (Sheldrick 2008)		
**Refinement method**	Full-matrix least-squares on F^2		
**Refinement program**	SHELXL-2014/7 (Sheldrick, 2014)		
**Function minimized**	Σ w(F_o^2 - F_c^2)^2		
**Data / restraints / parameters**	6766 / 0 / 376		
**Goodness-of-fit on F^2**	1.059		
**Final R indices**	5339 data;I>2σ(I)	R_1 = 0.0535	wR_2 = 0.1222
	all data	R_1 = 0.0721	wR_2 = 0.1345
**Weighting scheme**	w=1/[σ^2(F_o^2)+(0.0484P)^2+5.7516P] where P=(F_o^2+2F_c^2)/3		
**Largest diff. peak and hole**	1.864 and -0.996 eÅ^{-3}		

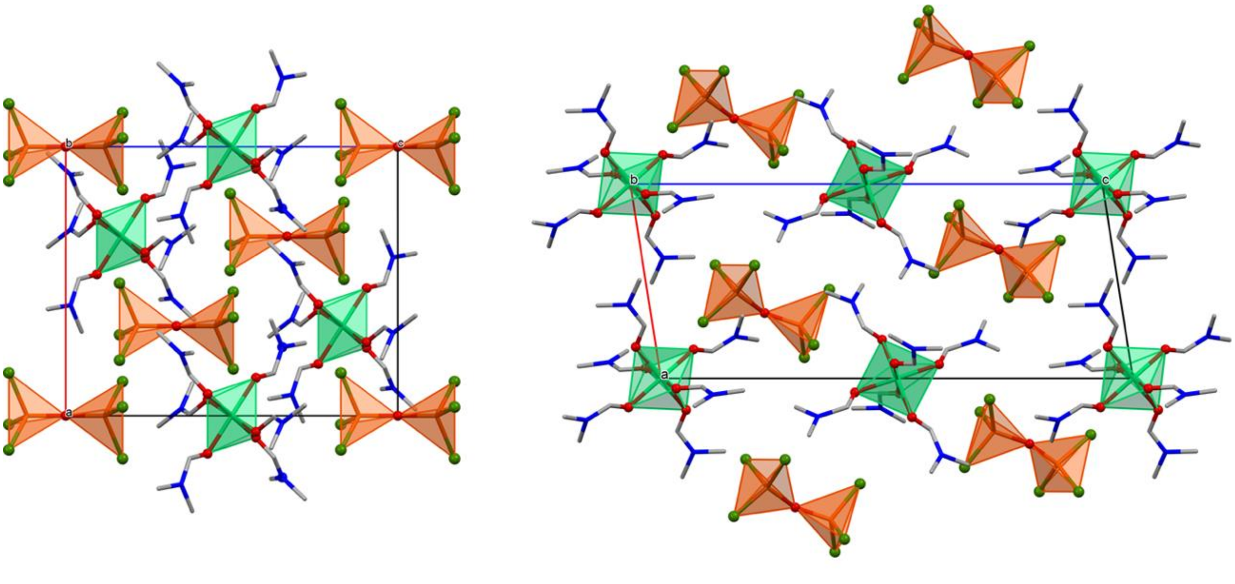

2A **2B**

Figure S4. View perpendicular to the *b* direction of the ion packing of the in the crystal structures **2A** (left) and **2B** (right).

d. Compound 3 (CCDC 1989768)

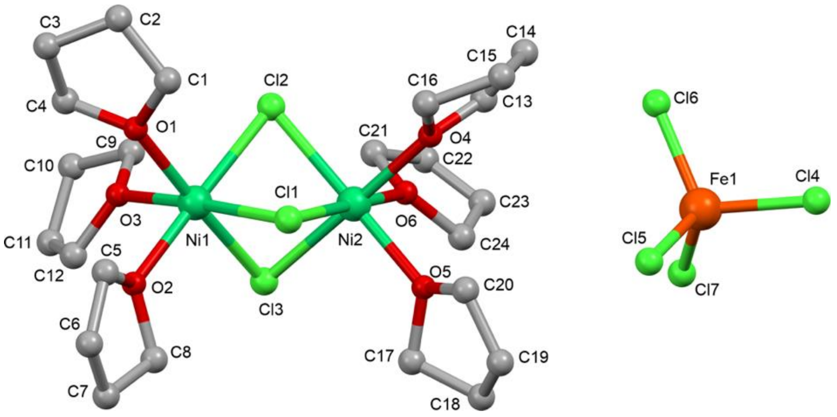

Figure S5. Asymmetric unit of compound **3** with atoms labelled.

Table S7. Sample and crystal data for **3.**

**CCDC Number**	1989768	
**Chemical formula**	$C_{12}H_4Cl_4FeNiS_4$	
**Formula weight**	532.77 g/mol	
**Temperature**	200(2) K	
**Wavelength**	0.71073 Å	
**Crystal size**	0.170 x 0.200 x 0.400 mm	
**Crystal habit**	yellow prismatic	
**Crystal system**	monoclinic	
**Space group**	$P2_1/c$	
**Unit cell dimensions**	a = 12.999(1) Å	α = 90°
	b = 12.1054(8) Å	β = 90.899(3)°
	c = 23.509 (2) Å	γ = 90°
**Volume**	1851.11(11) $Å^3$	
**Z**	4	
**Density (calculated)**	1.534 g/cm^3	
**Absorption coefficient**	1.933 mm^{-1}	
**F(000)**	1764	

Table S8. Data collection and structure refinement for **3.**

**Theta range for data collection**	1.57 to 25.42°		
**Index ranges**	-14<=h<=15, -14<=k<=14, -28<=l<=28		
**Reflections collected**	25236		
**Independent reflections**	6790 [R(int) = 0.0480]		
**Coverage of independent reflections**	99.4%		
**Absorption correction**	multi-scan		
**Max. and min. transmission**	0.7350 and 0.5120		
**Refinement method**	Full-matrix least-squares on F^2		
**Refinement program**	SHELXL-2014/7 (Sheldrick, 2014)		
**Function minimized**	$\Sigma\ w(F_o^2 - F_c^2)^2$		
**Data / restraints / parameters**	6790 / 0 / 361		
**Goodness-of-fit on F^2**	1.119		
**Final R indices**	4916 data;I>2σ(I)	R_1 = 0.0569	wR_2 = 0.1663
	all data	R_1 = 0.0850	wR_2 = 0.1906
**Weighting scheme**	$w=1/[\sigma^2(F_o^2)+(0.0968P)^2+5.1217P]$ where $P=(F_o^2+2F_c^2)/3$		
**Largest diff. peak and hole**	1.619 and -0.730 $eÅ^{-3}$		

e. Compound 4 (CCDC 1989769)

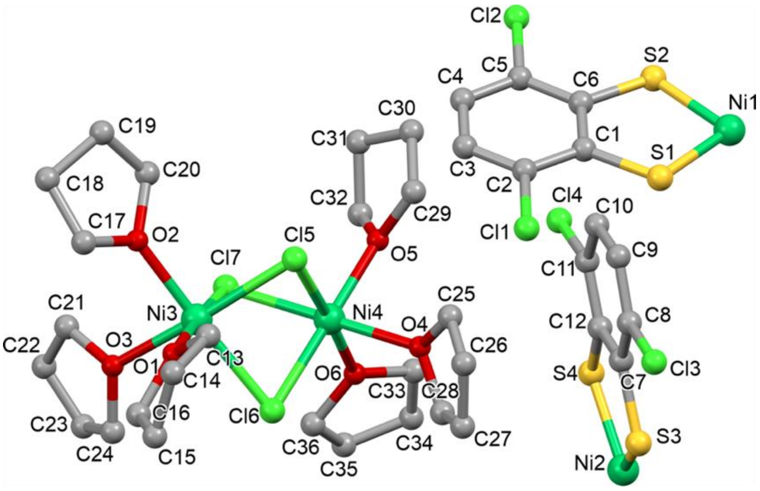

Figure S6. Asymmetric unit of compound **4** with atoms labelled.

Table S9. Sample and crystal data for **4.**

**CCDC Number**	1989769	
**Chemical formula**	$C_{36}H_{52}Cl_7Ni_3O_6S_4$	
**Formula weight**	1133.29 g/mol	
**Temperature**	200(2) K	
**Wavelength**	0.71073 Å	
**Crystal size**	0.120 x 0.200 x 0.264 mm	
**Crystal habit**	clear intense green prismatic	
**Crystal system**	triclinic	
**Space group**	*P*-1	
**Unit cell dimensions**	*a* = 14.2853(5)Å	α = 96.762(2)°
	b = 14.2853(5)Å	β = 90.792(2)°
	c = 18.0305(6)Å	γ = 109.046 (2)°
**Volume**	1851.11(11) Å^3	
**Z**	2	
**Density (calculated)**	1.598 g/cm^3	
**Absorption coefficient**	1.802 mm^{-1}	
**F(000)**	1166	

Table S10. Data collection and structure refinement for **4.**

**Theta range for data collection**	1.14 to 25.35°		
**Index ranges**	-11<=h<=11, -17<=k<=17, -21<=l<=21		
**Reflections collected**	80605		
**Independent reflections**	8617 [R(int) = 0.0398]		
**Coverage of independent reflections**	99.9%		
**Absorption correction**	multi-scan		
**Max. and min. transmission**	0.8130 and 0.6480		
**Structure solution technique**	direct methods		
**Structure solution program**	SHELXS-97 (Sheldrick 2008)		
**Refinement method**	Full-matrix least-squares on F^2		
**Refinement program**	SHELXL-2014/7 (Sheldrick, 2014)		
**Function minimized**	$\Sigma\ w(F_o^2 - F_c^2)^2$		
**Data / restraints / parameters**	8617 / 160 / 508		
**Goodness-of-fit on F^2**	1.070		
**Final R indices**	7383 data;I>2σ(I)	R_1 = 0.0326	wR_2 = 0.0834
	all data	R_1 = 0.0412	wR_2 = 0.0913
**Weighting scheme**	$w=1/[\sigma^2(F_o^2)+(0.0415P)^2+2.4517P]$ where $P=(F_o^2+2F_c^2)/3$		
**Largest diff. peak and hole**	0.867 and -0.488 eÅ^{-3}		

f. Compound 5 (CCDC 1989770)

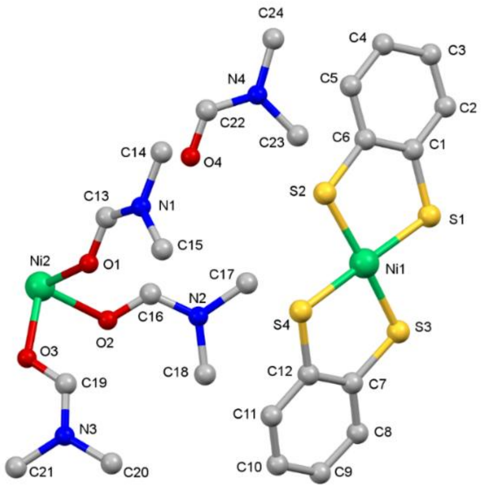

Figure S7. Asymmetric unit of compound **5** with atoms labelled.

Table S11. Sample and crystal data for **5.**

**CCDC Number**	1989770	
**Chemical formula**	$C_{48}H_{72}N_8Ni_3O_8S_8$	
**Formula weight**	1321.74 g/mol	
**Temperature**	200(2) K	
**Wavelength**	0.71073 Å	
**Crystal size**	0.020 x 0.050 x 0.140 mm	
**Crystal habit**	intense green plate	
**Crystal system**	monoclinic	
**Space group**	$P2_1/c$	
**Unit cell dimensions**	a = 8.4611(4)Å	α = 90°
	b = 18.882 (1)Å	β = 90.197(3)°
	c = 19.522(1)Å	γ = 90°
**Volume**	3118.9(3) $Å^3$	
**Z**	2	
**Density (calculated)**	1.407 g/cm^3	
**Absorption coefficient**	1.217 mm^{-1}	
**F(000)**	1384	

Table S12. Data collection and structure refinement for **5.**

**Theta range for data collection**	1.50 to 25.35°		
**Index ranges**	-10<=h<=10, -22<=k<=22, -23<=l<=23		
**Reflections collected**	54423		
**Independent reflections**	5714 [R(int) = 0.0624]		
**Coverage of independent reflections**	100.0%		
**Absorption correction**	multi-scan		
**Max. and min. transmission**	0.9760 and 0.8480		
**Structure solution technique**	direct methods		
**Structure solution program**	SHELXS-97 (Sheldrick 2008)		
**Refinement method**	Full-matrix least-squares on F^2		
**Refinement program**	SHELXL-2014/7 (Sheldrick, 2014)		
**Function minimized**	$\Sigma w(F_o^2 - F_c^2)^2$		
**Data / restraints / parameters**	5714 / 0 / 348		
**Goodness-of-fit on F^2**	1.086		
**Final R indices**	4328 data;I>2σ(I)	R_1 = 0.0297	wR_2 = 0.0688
	all data	R_1 = 0.0528	wR_2 = 0.0859
**Weighting scheme**	$w=1/[\sigma^2(F_o^2)+(0.0445P)^2]$where $P=(F_o^2+2F_c^2)/3$		
**Largest diff. peak and hole**	0.326 and -0.326 $eÅ^{-3}$		

g. Compound 6 (CCDC 1989771)

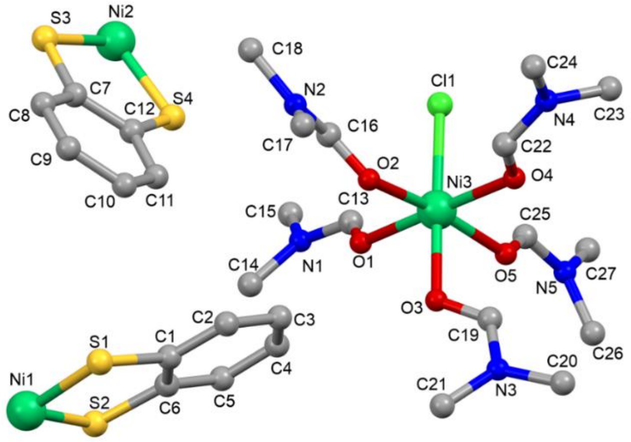

Figure S8. Asymmetric unit of compound **6** with atoms labelled.

Table S13. Sample and crystal data for **6.**

**CCDC Number**	1989771	
**Chemical formula**	$C_{27}H_{43}ClN_5Ni_2O_5S_4$	
**Formula weight**	798.77 g/mol	
**Temperature**	200(2) K	
**Wavelength**	0.71073 Å	
**Crystal size**	0.046 x 0.100 x 0.302 mm	
**Crystal habit**	clear intense green prismatic	
**Crystal system**	triclinic	
**Space group**	*P*-1	
**Unit cell dimensions**	*a* = 8.718(1)Å	α = 101.414(4)°
	b = 12.212 (1)Å	β = 97.572(4)°
	c = 17.889 (2)Å	γ = 99.593(5)°
**Volume**	1813.8(4) Å^3	
**Z**	2	
**Density (calculated)**	1.463 g/cm^3	
**Absorption coefficient**	1.383 mm^{-1}	
**F(000)**	834	

Table S14. Data collection and structure refinement for **6.**

**Theta range for data collection**	1.18 to 25.32°		
**Index ranges**	-10<=h<=10, -14<=k<=14, -21<=l<=21		
**Reflections collected**	32071		
**Independent reflections**	6624 [R(int) = 0.0443]		
**Coverage of independent reflections**	99.8%		
**Absorption correction**	multi-scan		
**Max. and min. transmission**	0.9390 and 0.6800		
**Structure solution technique**	direct methods		
**Structure solution program**	SHELXS-97 (Sheldrick 2008)		
**Refinement method**	Full-matrix least-squares on F^2		
**Refinement program**	SHELXL-2014/7 (Sheldrick, 2014)		
**Function minimized**	Σ w(F_o^2 - F_c^2)^2		
**Data / restraints / parameters**	6624 / 0 / 410		
**Goodness-of-fit on F^2**	1.091		
**Final R indices**	5286 data;I>2σ(I)	R_1 = 0.0312	wR_2 = 0.0758
	all data	R_1 = 0.0458	wR_2 = 0.0978
**Weighting scheme**	$w=1/[\sigma^2(F_o^2)+(0.0473P)^2+0.5309P]$where $P=(F_o^2+2F_c^2)/3$		
**Largest diff. peak and hole**	0.333 and -0.309 eÅ^{-3}		

h. Compound 7 (CCDC 1989772)

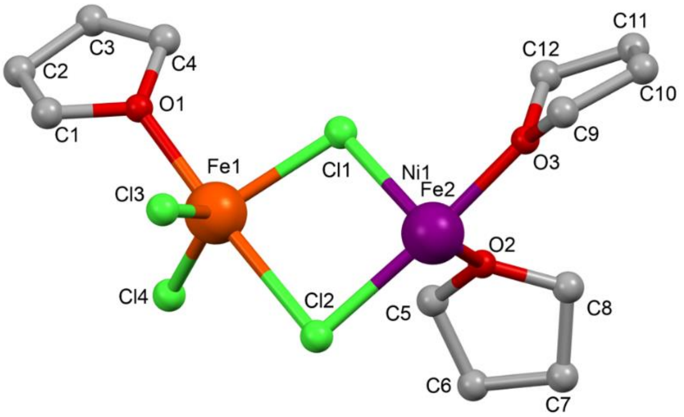

Figure S9. Asymmetric unit of compound **7** with atoms labelled.

Table S15. Sample and crystal data for **7.**

**CCDC Number**	1989772	
**Chemical formula**	$C_{24}H_{48}Cl_8Fe_{2.6}Ni_{1.4}O_6$	
**Formula weight**	943.60 g/mol	
**Temperature**	200(2) K	
**Wavelength**	0.71073 Å	
**Crystal size**	0.230 x 0.270 x 0.280 mm	
**Crystal system**	triclinic	
**Space group**	*P*-1	
**Unit cell dimensions**	*a* = 9.9511(6) Å	α = 62.650(2)°
	b = 10.4813(5) Å	β = 67.991(3)°
	c = 10.8907(7) Å	γ = 82.248(3)°
**Volume**	934.4(1) $Å^3$	
**Z**	1	
**Density (calculated)**	1.677 g/cm^3	
**Absorption coefficient**	2.290 mm^{-1}	
**F(000)**	485.5	

Table S16. Data collection and structure refinement for **7.**

**Theta range for data collection**	2.53 to 25.41°		
**Index ranges**	-11<=h<=11, -12<=k<=12, -13<=l<=12		
**Reflections collected**	14885		
**Independent reflections**	3419 [R(int) = 0.0369]		
**Absorption correction**	multi-scan		
**Max. and min. transmission**	0.6210 and 0.5670		
**Refinement method**	Full-matrix least-squares on F^2		
**Refinement program**	SHELXL-2014/7 (Sheldrick, 2014)		
**Function minimized**	Σ w(F_o^2 - F_c^2)^2		
**Data / restraints / parameters**	3419 / 0 / 190		
**Goodness-of-fit on F^2**	1.031		
**Final R indices**	2948 data;I>2σ(I)	R_1 = 0.0310	wR_2 = 0.0772
	all data	R_1 = 0.0376	wR_2 = 0.0824
**Weighting scheme**	w=1/[σ^2(F_o^2)+(0.0333P)^2+1.2619P] where P=(F_o^2+2F_c^2)/3		
**Largest diff. peak and hole**	0.400 and -0.470 e$Å^{-3}$		

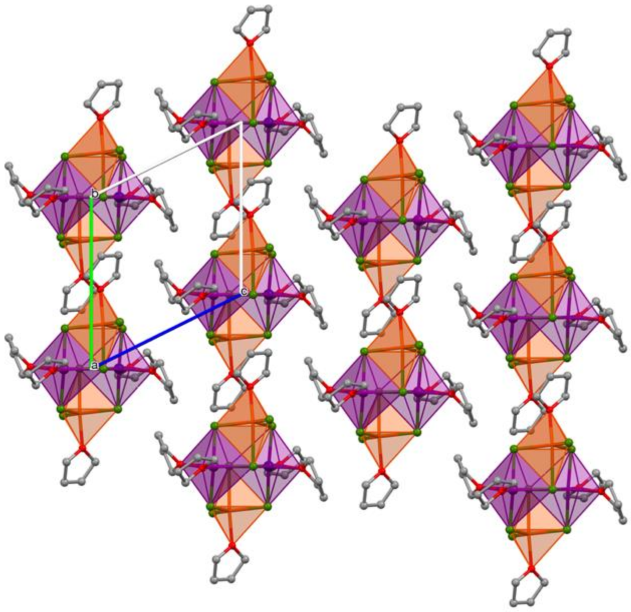

Figure S10. View in the *a* direction of the packing of the tetrametallic molecules of compound **7** in its crystal structure. In orange are represented the coordination polyhedra around the positions occupied only by iron atoms (Fe1), and in purple, the polyhedra for the metal positions shared by Fe and Ni atoms (Fe2/Ni1).

S2. Metal coordination environment parameters

Table S17. Metal coordination bond distances (Å) for compounds **1-7.**

**1**	Ni1—S1	2.136 (2)
	Ni1—S2	2.143 (2)
	Ni1—S3	2.146 (2)
	Ni1—S4	2.138 (2)
	Ni2—O1^{i}	2.035 (3)
	Ni2—O1	2.035 (3)
	Ni2—O2	2.065 (4)
	Ni2—O2^{i}	2.065 (4)
	Ni2—O3	2.051 (3)
	Ni2—O3^{i}	2.052 (3)
	(i) −*x*, −*y*+1, −*z*	
**2A**	Fe1—Cl1	2.231(1)
	Fe1—Cl1^{i}	2.231(1)
	Fe1—Cl1^{ii}	2.231(1)
	Fe1—O2	1.7663(9)
	Ni1—O1	2.058 (2)
	Ni1—O1^{i}	2.058 (2)
	Ni1—O1^{ii}	2.058 (2)
	Ni1—O1^{iii}	2.058(2)
	Ni1—O1^{iv}	2.058(2)
	Ni1—O1^{v}	2.058 (2)
	(i) −y+1, x−y+1, z; (ii) −x+y, −x+1, z; (iii) x−y+2/3, x+1/3, −z+1/3; (iv) y−1/3, −x+y+1/3,	
**2B**	Fe1—O7	1.753 (3)
	Fe1—Cl1	2.222 (1)
	Fe1—Cl2	2.220 (1)
	Fe1—Cl3	2.219 (2)
	Fe2—Cl4	2.220 (2)
	Fe2—O7	1.758 (3)
	Fe2—Cl5	2.221(2)
	Fe2—Cl6	2.223(2)
	Ni1—O1	2.052 (4)
	Ni1—O1^{i}	2.052 (4)
	Ni1—O2^{i}	2.043 (3)
	Ni1—O2	2.043 (3)
	Ni1—O3^{i}	2.051 (3)
	Ni1—O3	2.052 (3)
	Ni2—O4^{ii}	2.067 (5)
	Ni2—O4	2.067 (5)
	Ni2—O5^{ii}	2.082 (5)
	Ni2—O5	2.082 (5)
	Ni2—O6^{ii}	2.034 (4)
	Ni2—O6	2.034 (4)
	(i) −x+2, −y+2, −z; (ii) −x, −y, −z+1	
**3**	Ni1—O1	2.074 (4)
	Ni1—O2	2.092 (4)
	Ni1—O3	2.091 (4)
	Ni1—Cl1	2.415 (2)
	Ni1—Cl2	2.423 (2)
	Ni1—Cl3	2.390 (2)
	Ni2—O4	2.072 (4)
	Ni2—O5	2.070 (4)
	Ni2—O6	2.104 (4)
	Ni2—Cl1	2.439 (2)

	Ni2—Cl2	2.402 (2)
	Ni2—Cl3	2.395 (2)
	Fe1—Cl4	2.184 (2)
	Fe1—Cl5	2.160 (3)
	Fe1—Cl6	2.156 (2)
	Fe1—Cl7	2.176 (3)
	Ni2—Ni1	2.976 (1)
**4**	Ni1—S1	2.1521 (8)
	Ni1—S1^{i}	2.1521 (8)
	Ni1—S2	2.1389 (8)
	Ni1—S2^{i}	2.1389 (8)
	Ni2—S3^{ii}	2.1416 (7)
	Ni2—S3	2.1416 (7)
	Ni2—S4	2.1435 (8)
	Ni2—S4^{ii}	2.1435 (7)
	Ni3—O1	2.059 (2)
	Ni3—O2	2.065 (2)
	Ni3—O3	2.115 (2)
	Ni3—Cl5	2.4299 (9)
	Ni3—Cl6	2.4286 (8)
	Ni3—Cl7	2.4047 (8)
	Ni4—O6	2.089 (2)
	Ni4—O5	2.093 (2)
	Ni4—O4	2.095 (2)
	Ni4—Cl5	2.4002 (9)
	Ni4—Cl6	2.4133 (9)
	Ni4—Cl7	2.4053 (8)
	Ni3—Ni4	2.9849 (5)
	(i) −x+1, −y, −z+2; (ii) −x+1, −y, −z+1.	
**5**	Ni1—S1	2.1494 (7)
	Ni1—S2	2.1441 (8)
	Ni1—S3	2.1428 (7)
	Ni1—S4	2.1494 (8)
	Ni2—O1	2.054 (2)
	Ni2—O1^{i}	2.054 (2)
	Ni2—O2	2.055 (2)
	Ni2—O2^{i}	2.055 (2)
	Ni2—O3	2.030 (2)
	Ni2—O3^{i}	2.030 (2)
	(i) −x+2, −y+1, −z+1.	
**6**	Ni1—S1	2.1541 (8)
	Ni1—S1^{i}	2.1541 (8)
	Ni1—S2	2.1521 (8)
	Ni1—S2^{i}	2.1521 (8)
	Ni2—S3	2.1521 (9)
	Ni2—S3^{ii}	2.1521 (9)
	Ni2—S4	2.1569 (9)
	Ni2—S4^{ii}	2.1570 (9)
	Ni3—O1	2.065 (2)
	Ni3—O2	2.070 (2)
	Ni3—O3	2.107 (2)
	Ni3—O4	2.083 (2)
	Ni3—O5	2.038 (2)
	Ni3—Cl1	2.3723 (8)
	(ii) *−x, −y, −z.*	
**7**	Fe1—O1	2.130 (2)
	Fe1—Cl1	2.3600 (9)
	Fe1—Cl2	2.7018 (8)
	Fe1—Cl3	2.3565 (9)
	Fe1—Cl4	2.252 (1)

	Ni1/Fe2—O2	2.115 (2)
	Ni1/Fe2—O3	2.087 (2)
	Ni1/Fe2—Cl1	2.4375 (8)
	Ni1/Fe2—Cl2	2.4657 (8)
	Ni1/Fe2—Cl2^{i}	2.4814 (8)
	Ni1/Fe2—Cl3^{i}	2.4491 (8)
	(i) −x+1, −y+2, −z+2.	

Table S18. Selected metal coordination bond angles (°) for compounds **1-7.**

**1**	S1—Ni1—S4	176.62 (6)
	S1—Ni1—S2	92.16 (6)
	S4—Ni1—S2	87.05 (6)
	S1—Ni1—S3	88.84 (6)
	S4—Ni1—S3	92.14 (6)
	S2—Ni1—S3	176.76 (6)
	O1^{i}—Ni2—O1	180.0 (2)
	O1^{i}—Ni2—O3	88.8 (1)
	O1—Ni2—O3	91.2 (1)
	O1^{i}—Ni2—O3^{i}	91.2 (1)
	O1—Ni2—O3^{i}	88.8 (1)
	O3—Ni2—O3^{i}	180
	O1^{i}—Ni2—O2	91.5 (1)
	O1—Ni2—O2	88.5 (1)
	O3—Ni2—O2	90.2 (1)
	O3^{i}—Ni2—O2	89.8 (1)
	O1^{i}—Ni2—O2^{i}	88.5 (1)
	O1—Ni2—O2^{i}	91.5 (1)
	O3—Ni2—O2^{i}	89.8 (1)
	O3^{i}—Ni2—O2^{i}	90.2 (1)
	O2—Ni2—O2^{i}	180
	(i) −x, −y+1, −z.	
**2A**	O2—Fe1—Cl1^{i}	111.78 (3)
	O2—Fe1—Cl1^{ii}	111.78 (3)
	Cl1^{i}—Fe1—Cl1^{ii}	107.07 (4)
	O2—Fe1—Cl1	111.78 (3)
	Cl1^{i}—Fe1—Cl1	107.07 (4)
	Cl1^{ii}—Fe1—Cl1	107.07 (4)
	O1^{iii}—Ni1—O1^{iv}	93.98 (9)
	O1^{iii}—Ni1—O1^{i}	86.02 (9)
	O1^{iv}—Ni1—O1^{i}	180
	O1^{iii}—Ni1—O1^{ii}	180
	O1^{iv}—Ni1—O1^{ii}	86.03 (9)
	O1^{i}—Ni1—O1^{ii}	93.98 (9)
	O1^{iii}—Ni1—O1	86.03 (9)
	O1^{iv}—Ni1—O1	86.03 (9)
	O1^{i}—Ni1—O1	93.97 (9)
	O1^{ii}—Ni1—O1	93.97 (9)
	O1^{iii}—Ni1—O1^{v}	93.97 (9)
	O1^{iv}—Ni1—O1^{v}	93.97 (9)
	O1^{i}—Ni1—O1^{v}	86.03 (9)
	O1^{ii}—Ni1—O1^{v}	86.02 (9)
	O1—Ni1—O1^{v}	180
	(i) −y+1, x−y+1, z; (ii) −x+y, −x+1, z; (iii) x−y+2/3, x+1/3, −z+1/3; (iv) y−1/3,	
**2B**	O1—Ni1—O1^{i}	180
	O1—Ni1—O2	90.2 (1)

	O1—Ni1—O2^{i}	89.8 (1)
	O1—Ni1—O3	89.7 (1)
	O1—Ni1—O3^{i}	90.3 (1)
	O1^{i}—Ni1—O2^{i}	90.2 (1)
	O1^{i}—Ni1—O3^{i}	89.7 (1)
	O2—Ni1—O1^{i}	89.8 (1)
	O2—Ni1—O3	90.3 (1)
	O2—Ni1—O3^{i}	89.7 (1)
	O2^{i}—Ni1—O3	89.7 (1)
	O2^{i}—Ni1—O3^{i}	90.3 (1)
	O3—Ni1—O1^{i}	90.3 (1)
	O3—Ni1—O3^{i}	180
	O4—Ni2—O4^{ii}	180
	O4—Ni2—O5	89.1 (2)
	O4—Ni2—O5^{ii}	90.9 (2)
	O4—Ni2—O6	91.9 (2)
	O4—Ni2—O6^{ii}	88.1 (2)
	O4^{ii}—Ni2—O5^{ii}	89.1 (2)
	O4^{ii}—Ni2—O6^{ii}	91.9 (2)
	O5—Ni2—O4^{ii}	90.9 (2)
	O5—Ni2—O5^{ii}	180
	O5—Ni2—O6	88.8 (2)
	O5—Ni2—O6^{ii}	91.3 (2)
	O5^{ii}—Ni2—O6	91.3 (2)
	O5^{ii}—Ni2—O6^{ii}	88.8 (2)
	O6—Ni2—O4^{ii}	88.1 (2)
	O6—Ni2—O6^{ii}	180
	Cl1—Fe1—Cl2	109.68 (6)
	Cl1—Fe1—Cl3	106.48 (6)
	Cl1—Fe1—O7	110.6 (1)
	Cl2—Fe1—Cl3	109.08 (6)
	Cl2—Fe1—O7	110.5 (1)
	Cl3—Fe1—O7	110.4 (1)
	Cl4—Fe2—Cl5	106.93 (7)
	Cl4—Fe2—Cl6	109.06 (7)
	Cl4—Fe2—O7	111.6 (1)
	Cl5—Fe2—Cl6	110.47 (8)
	Cl5—Fe2—O7	110.0 (1)
	Cl6—Fe2—O7	108.7 (13)
	Fe1—O7—Fe2	154.2 (2)
	(i) −x+2, −y+2, −z; (ii) −x, −y, −z+1.	
**3**	O1—Ni1—O2	87.9 (2)
	O1—Ni1—O3	88.4 (2)
	O1—Ni1—Cl1	96.0 (1)
	O1—Ni1—Cl2	89.9 (1)
	O1—Ni1—Cl3	175.5 (2)
	O2—Ni1—O3	90.0 (2)
	O2—Ni1—Cl1	89.7 (1)
	O2—Ni1—Cl2	14.0 (1)
	O2—Ni1—Cl3	96.0 (1)
	O3—Ni1—Cl1	175.6 (1)
	O3—Ni1—Cl2	95.6 (1)
	O3—Ni1—Cl3	89.3 (1)
	Cl1—Ni1—Cl2	84.97 (6)
	Cl1—Ni1—Cl3	86.33 (6)
	Cl2—Ni1—Cl3	86.50 (6)
	O4—Ni2—O5	87.0 (2)
	O4—Ni2—O6	88.5 (2)
	O4—Ni2—Cl1	94.4 (1)
	O4—Ni2—Cl2	92.0 (1)

	O4—Ni2—Cl3	178.9 (1)
	O5—Ni2—O6	89.1 (2)
	O5—Ni2—Cl1	91.9 (1)
	O5—Ni2—Cl2	176.5 (1)
	O5—Ni2—Cl3	94.1 (1)
	O6—Ni2—Cl1	177.0 (1)
	O6—Ni2—Cl2	94.2 (1)
	O6—Ni2—Cl3	91.39 (12)
	Cl1—Ni2—Cl2	84.91 (6)
	Cl1—Ni2—Cl3	85.69 (6)
	Cl2—Ni2—Cl3	86.88 (6)
	Ni1—Cl1—Ni2	75.64 (5)
	Ni1—Cl2—Ni2	76.17 (5)
	Ni1—Cl3—Ni2	76.92 (5)
	Cl4—Fe1—Cl5	108.8 (1)
	Cl4—Fe1—Cl6	109.7 (1)
	Cl4—Fe1—Cl7	110.5 (1)
	Cl5—Fe1—Cl6	111.3 (2)
	Cl5—Fe1—Cl7	108.5 (2)
	Cl6—Fe1—Cl7	108.1 (1)
4	S2—Ni1—S2^{i}	180.0
	S2—Ni1—S1	92.12 (3)
	S2^{i}—Ni1—S1	87.88 (3)
	S2—Ni1—S1^{i}	87.88 (3)
	S2^{i}—Ni1—S1^{i}	92.12 (3)
	S1—Ni1—S1^{i}	180
	S3^{ii}—Ni2—S3	180
	S3^{ii}—Ni2—S4	87.71 (3)
	S3—Ni2—S4	92.29 (3)
	S3^{ii}—Ni2—S4^{ii}	92.29 (3)
	S3—Ni2—S4^{ii}	87.71 (3)
	S4—Ni2—S4^{ii}	180
	O1—Ni3—O2	86.19 (8)
	O1—Ni3—O3	90.26 (8)
	O2—Ni3—O3	87.42 (8)
	O1—Ni3—Cl7	175.68 (6)
	O2—Ni3—Cl7	97.79 (6)
	O3—Ni3—Cl7	88.26 (6)
	O1—Ni3—Cl6	89.38 (6)
	O2—Ni3—Cl6	173.62 (7)
	O3—Ni3—Cl6	97.21 (6)
	Cl7—Ni3—Cl6	86.79 (3)
	O1—Ni3—Cl5	96.34 (6)
	O2—Ni3—Cl5	91.63 (6)
	O3—Ni3—Cl5	173.26 (6)
	Cl7—Ni3—Cl5	85.26 (3)
	Cl6—Ni3—Cl5	84.29 (3)
	O1—Ni3—Ni4	126.70 (6)
	O2—Ni3—Ni4	128.50 (6)
	O3—Ni3—Ni4	124.93 (6)
	Cl7—Ni3—Ni4	51.65 (2)
	Cl6—Ni3—Ni4	51.71 (2)
	Cl5—Ni3—Ni4	51.38 (2)
	O6—Ni4—O5	86.83 (10)
	O6—Ni4—O4	87.50 (10)
	O5—Ni4—O4	88.01 (11)
	O6—Ni4—Cl5	176.87 (7)
	O5—Ni4—Cl5	95.61 (7)
	O4—Ni4—Cl5	94.54 (8)
	O6—Ni4—Cl7	92.06 (7)

	O5—Ni4—Cl7	91.95 (8)
	O4—Ni4—Cl7	179.56 (8)
	Cl5—Ni4—Cl7	85.90 (3)
	O6—Ni4—Cl6	92.26 (7)
	O5—Ni4—Cl6	178.68 (7)
	O4—Ni4—Cl6	92.91 (8)
	Cl5—Ni4—Cl6	85.27 (3)
	Cl7—Ni4—Cl6	87.12 (3)
	O6—Ni4—Ni3	124.61 (7)
	O5—Ni4—Ni3	127.75 (8)
	O4—Ni4—Ni3	128.69 (8)
	Cl5—Ni4—Ni3	52.28 (2)
	Cl7—Ni4—Ni3	51.63 (2)
	Cl6—Ni4—Ni3	52.17 (2)
	(i) −x+1, −y, −z+2; (ii) −x+1, −y, −z+1.	
**5**	S1—Ni1—S2	92.05 (3)
	S1—Ni1—S3	88.08 (3)
	S1—Ni1—S4	178.32 (3)
	S2—Ni1—S3	173.56 (3)
	S2—Ni1—S4	88.28 (3)
	S3—Ni1—S4	91.78 (3)
	O1—Ni2—O1^{i}	180
	O1—Ni2—O2	87.32 (7)
	O1—Ni2—O2^{i}	92.68 (7)
	O1—Ni2—O3	89.53 (6)
	O1—Ni2—O3^{i}	90.47 (6)
	O1^{i}—Ni2—O2^{i}	87.32 (7)
	O1^{i}—Ni2—O3^{i}	89.53 (6)
	O2—Ni2—O1^{i}	92.68 (7)
	O2—Ni2—O3	88.09 (7)
	O2—Ni2—O2^{i}	180.0
	O2—Ni2—O3^{i}	91.91 (7)
	O2^{i}—Ni2—O3^{i}	88.09 (7)
	O3—Ni2—O1^{i}	90.47 (6)
	O3—Ni2—O2^{i}	91.91 (7)
	O3—Ni2—O3^{i}	180.0
	(i) −x+2, −y+1, −z+1.	
**6**	S1—Ni1—S2	92.05 (3)
	S1—Ni1—S1^{i}	180
	S1—Ni1—S2^{i}	87.95 (3)
	S1^{i}—Ni1—S2^{i}	92.05 (3)
	S2—Ni1—S1^{i}	87.95 (3)
	S2—Ni1—S2^{i}	180
	S3—Ni2—S4	91.76 (3)
	S3—Ni2—S3^{ii}	180.00 (4)
	S3—Ni2—S4^{ii}	88.24 (3)
	S3^{ii}—Ni2—S4^{ii}	91.76 (3)
	S4—Ni2—S3^{ii}	88.24 (3)
	S4—Ni2—S4^{ii}	180
	O1—Ni3—O2	89.94 (8)
	O1—Ni3—O3	90.43 (7)
	O1—Ni3—O4	179.33 (8)
	O1—Ni3—O5	91.92 (8)
	O1—Ni3—Cl1	92.92 (6)
	O2—Ni3—O3	85.11 (8)
	O2—Ni3—O4	90.49 (8)
	O2—Ni3—O5	169.95 (8)
	O2—Ni3—Cl1	93.00 (6)
	O3—Ni3—O4	89.10 (7)
	O3—Ni3—O5	85.00 (8)

	O3—Ni3—Cl1	176.16 (6)
	O4—Ni3—O5	87.56 (8)
	O4—Ni3—Cl1	87.57 (6)
	O5—Ni3—Cl1	96.77 (6)
	(i) −x+1, −y, −z+1; (ii) −x, −y, −z.	
**7**	O1—Fe1—Cl1	92.82 (7)
	O1—Fe1—Cl2	173.23 (7)
	O1—Fe1—Cl3	90.63 (7)
	O1—Fe1—Cl4	94.62 (7)
	Cl1—Fe1—Cl2	84.67 (3)
	Cl1—Fe1—Cl3	108.50 (3)
	Cl1—Fe1—Cl4	124.47 (4)
	Cl2—Fe1—Cl3	84.24 (3)
	Cl2—Fe1—Cl4	91.98 (3)
	Cl3—Fe1—Cl4	126.32 (4)
	O2— Ni1/Fe2—O3	88.39 (9)
	O2—Ni1/Fe2—Cl1	87.72 (6)
	O2—Ni1/Fe2—Cl2	95.09 (7)
	O2—Ni1/Fe2—$Cl2^{i}$	176.43 (7)
	O2—Ni1/Fe2—$Cl3^{i}$	89.60 (6)
	O3—Ni1/Fe2—Cl1	89.39 (6)
	O3—Ni1/Fe2—Cl2	175.79 (7)
	O3—Ni1/Fe2—$Cl2^{i}$	93.25 (6)
	O3—Ni1/Fe2—$Cl3^{i}$	88.79 (6)
	Cl1—Ni1/Fe2—Cl2	88.39 (3)
	Cl1—Ni1/Fe2—$Cl2^{i}$	95.47 (3)
	Cl1—Ni1/Fe2—$Cl3^{i}$	176.80 (3)
	Cl2—Ni1/Fe2—$Cl2^{i}$	83.41 (3)
	Cl2—Ni1/Fe2—$Cl3^{i}$	93.59 (3)
	$Cl2^{i}$—Ni1/Fe2—$Cl3^{i}$	87.26 (3)
	(i) −x+1, −y+2, −z+2.	

S3. Supramolecular interactions

Table S19. Supramolecular interactions (Å,°) for compounds **1-7.**

	D—H···*A*	*D*—H	H···*A*	*D*···*A*	*D*—H···*A*
**1**	C4—H4···S4^{ii}	0.95	3.01	3.771 (4)	138
	C15—H15B···S4^{iii}	0.98	2.93	3.713 (4)	138
	C16—H16···O1^{i}	0.95	2.55	3.040 (5)	113
	C18—H18C···O2^{iv}	0.98	2.64	3.550 (5)	155
	C18—H18C···O3^{v}	0.98	2.61	3.368 (6)	135
	C19—H19···O1	0.95	2.58	3.082 (5)	113
	(i) −x, −y+1, −z; (ii) −x+1/2, y−1/2, −z+1/2; (iii) −x+1/2, y+1/2, −z+1/2; (iv) −x+1, −y+1, −z; (v)				
**2A**	C1—H1···O1^{ii}	0.95	2.50	3.078 (4)	119
	C2—H2C···Cl1^{vii}	0.98	2.96	3.822 (4)	148
	C3—H3B···Cl1^{vii}	0.98	2.96	3.859 (4)	153
	(ii) −x+y, −x+1, z; (vii) −x+1, −y+1, −z+1.				
**2B**	C1—H1···O2	0.95	2.57	3.044 (6)	111
	C2—H2C···Cl6^{iii}	0.98	2.97	3.875 (9)	153
	C3—H3A···Cl4	0.98	2.72	3.607 (8)	152
	C5—H5C···Cl1	0.98	2.83	3.780 (7)	163
	C6—H6C···Cl6^{iv}	0.98	2.89	3.855 (7)	168
	C7—H7···O2	0.95	2.48	3.020 (6)	116
	C11—H11A···Cl4^{v}	0.98	3.01	3.495 (9)	112
	C11—H11B···Cl3^{v}	0.98	2.78	3.676 (9)	153
	C12—H12A···Cl6	0.98	2.77	3.702 (7)	158
	C13—H13···O4	0.95	2.34	2.849 (10)	113
	C14—H14C···Cl3	0.98	2.88	3.753 (6)	149
	(iii) −x+1, −y+1, −z; (iv) x, y+1, z; (v) x, y−1, z.				
**3**	C20—H20A···Cl1	0.99	2.96	3.558 (8)	120
	C19—H19B···Cl5	0.99	2.93	3.610 (9)	127
	C18—H18B···Cl4^{i}	0.99	2.87	3.749 (9)	148
	C17—H17A···Cl3^{ii}	0.99	2.82	3.561 (8)	132
	C16—H16A···Cl1	0.99	2.83	3.338 (8)	113
	C13—H13B···Cl6	0.99	2.94	3.636 (10)	128
	C7—H7A···Cl7^{ii}	0.99	2.85	3.625 (11)	135
	C4—H4B···Cl6^{iii}	0.99	2.89	3.763 (11)	148
	C1—H1A···Cl2	0.99	2.94	3.529 (8)	119
	(i) −x+1, y−1/2, −z+1/2; (ii) −x+1, −y+1, −z+1; (iii) −x+2, −y+1, −z+1				
**4**	C9—H9···S1	0.95	2.87	3.810 (3)	170
	C15—H15A···Cl3^{iii}	0.99	2.90	3.784 (4)	149
	C18—H18B···Cl3^{iv}	0.99	2.92	3.750 (4)	143

	C19—H19A···S2^{v}	0.99	2.92	3.670 (4)	133
	C20—H20B···Cl5	0.99	2.93	3.531 (4)	120
	C21—H21A···Cl4^{iii}	0.99	2.93	3.891 (4)	165
	C21—H21B···Cl7	0.99	2.81	3.404 (4)	119
	C24—H24B···Cl6	0.99	2.70	3.449 (4)	133
	C25—H25B···Cl5	0.99	2.64	3.376 (6)	132
	C28—H28B···Cl6	0.99	2.85	3.466 (6)	121
	C29—H29B···Cl5	0.99	2.84	3.523 (5)	126
	C32—H32A···Cl7	0.99	2.64	3.338 (7)	128
	C36—H36A···Cl7	0.99	2.96	3.546 (4)	119
	(iii) x, y+1, z; (iv) x+1, y+1, z; (v) −x+2, −y+1, −z+2.				
**5**	C13—H13···O3^{i}	0.95	2.49	3.024 (3)	116
	C15—H15*B*···O2^{ii}	0.98	2.58	3.520 (3)	161
	C15—H15*C*···S4	0.98	2.90	3.858 (3)	167
	C16—H16···O3^{i}	0.95	2.56	3.103 (3)	116
	C17—H17*A*···O4	0.98	2.54	3.491 (4)	165
	C19—H19···O1	0.95	2.53	3.051 (3)	115
	C21—H21*A*···O4^{i}	0.98	2.58	3.495 (3)	156
	C21—H21*B*···S3^{iii}	0.98	2.90	3.539 (3)	124
	C21—H21*C*···S1^{iv}	0.98	2.95	3.824 (3)	149
	(i) −x+2, −y+1, −z+1; (ii) x−1, y, z; (iii) x, −y+1/2, z−1/2; (iv) x+1, −y+1/2, z−1/2.				
**6**	C13—H13···Cl1	0.95	2.71	3.263 (3)	118
	C13—H13···Cl1^{iii}	0.95	2.91	3.794 (3)	155
	C14—H14B···S3^{iv}	0.98	3.02	3.837 (4)	141
	C15—H15C···Cl1^{iii}	0.98	2.83	3.784 (3)	165
	C16—H16···Cl1	0.95	2.79	3.260 (3)	111
	C19—H19···O4	0.95	2.40	2.917 (4)	114
	C20—H20B···S2^{v}	0.98	2.84	3.692 (4)	146
	C22—H22···O2	0.95	2.39	2.952 (3)	118
	C24—H24C···S2^{vi}	0.98	2.97	3.622 (3)	125
	C25—H25···Cl1	0.95	2.82	3.417 (3)	122
	C25—H25···Cl1^{iii}	0.95	2.85	3.681 (3)	146
	C26—H26B···Cl1^{iv}	0.98	2.69	3.672 (4)	179
	C27—H27A···Cl1^{iii}	0.98	2.81	3.719 (3)	155
	(iii) −x+1, −y+1, −z; (iv) x+1, y, z; (v) −x+2, −y+1, −z+1; (vi) x, y+1, z.				
**7**	C3—H3B···Cl2^{ii}	0.99	2.90	3.827 (4)	157
	C5—H5A···Cl4^{iii}	0.99	2.93	3.699 (3)	135
	C5—H5B···Cl2	0.99	2.79	3.461 (4)	126
	C8—H8A···Cl3^{i}	0.99	2.77	3.347 (4)	118
	C9—H9A···Cl3^{i}	0.99	2.98	3.559 (4)	119
	(i) −x+1, −y+2, −z+2; (ii) x, y−1, z; (iii) −x+2, −y+2, −z+1				

S4. TXRF analyses for compounds 1, 2A and 3-7.

Table S20. TXRF data for the measured single crystal of **1**.

Element	Line	Conc./ umr	Sigma/ umr	RSD/ %	LLD/ umr	Net area	Backgr.	Chi
Si	K12	47.32	0.48	1.0	0.57	14388	3290	2.98
S	K12	68.87	0.26	0.4	0.17	76878	3789	3.09
Cl	K12	63.74	0.19	0.3	0.10	117134	3423	7.23
K	K12	0.194	0.020	10.5	0.041	779	2965	2.08
Fe	K12	0.538	0.007	1.2	0.010	12121	5091	1.52
Ni (IS)	K12	58.690	0.045	0.1	0.007	1927142	5577	31.49
Cu	K12	0.337	0.003	1.0	0.003	12956	1563	1.71
Pt	L1	0.032	0.001	3.9	0.002	1218	512	1.00

Table S21. TXRF data for the measured single crystal of **2A**.

Element	Line	Conc./ umr	Sigma/ umr	RSD/ %	LLD/ umr	Net area	Backgr.	Chi
Si	K12	414.9	4.5	1.1	4.1	11413	1437	2.70
S	K12	4.87	0.56	11.5	1.09	492	1337	0.75
Cl	K12	101.67	0.87	0.9	0.66	16904	1324	2.85
Ca	K12	0.33	0.11	33.5	0.22	143	1075	1.30
Fe	K12	115.51	0.28	0.2	0.06	235539	1693	2.71
Ni (IS)	K12	58.69	0.16	0.3	0.04	174372	1584	3.48
Cu	K12	4.745	0.042	0.9	0.033	16503	1437	1.52
Zn	K12	0.268	0.015	5.4	0.025	1090	1175	0.97
Br	K12	0.200	0.008	4.2	0.013	1288	783	1.21

Table S22. TXRF data for the measured single crystal of **3**.

Element	Line	Conc./ umr	Sigma/ umr	RSD/ %	LLD/ umr	Net area	Backgr.	Chi
Si	K12	331.6	3.1	0.9	2.7	15144	1672	2.63
Cl	K12	45.56	0.47	1.0	0.44	12578	1611	2.40
Mn	K12	2.087	0.041	2.0	0.061	5636	2989	27.91
Fe (IS)	K12	55.85	0.15	0.3	0.05	189079	3029	18.03
Ni	K12	94.24	0.18	0.2	0.05	464863	6635	47.86
Zn	K12	0.533	0.012	2.3	0.017	3598	1393	1.43

Table S23. TXRF data for the measured single crystal of **4**.

Element	Line	Conc./ umr	Sigma/ umr	RSD/ %	LLD/ umr	Net area	Backgr.	Chi
Si	K12	1038	14	1.4	11	7959	728	2.09
S	K12	59.0	2.1	3.5	2.9	1663	721	0.62
Cl	K12	95.6	1.8	1.9	1.7	4431	725	1.33
Fe (IS)	K12	55.85	0.41	0.7	0.19	31747	1238	2.33
Ni	K12	405.6	1.3	0.3	0.2	335889	3455	24.22
Zn	K12	1.131	0.042	3.7	0.054	1282	416	1.01

Table S24. TXRF data for the measured single crystal of **5**.

Element	Line	Conc./ umr	Sigma/ umr	RSD/ %	LLD/ umr	Net area	Backgr.	Chi
Si	K12	23.02	0.29	1.3	0.40	10341	3542	1.09
S	K12	56.62	0.19	0.3	0.11	93410	3522	3.22
Fe	K12	0.218	0.004	2.0	0.007	7273	6727	3.91
Ni (IS)	K12	58.690	0.037	0.1	0.005	2847805	7356	67.53
Cu	K12	0.644	0.004	0.6	0.004	36593	6269	3.87
Zn	K12	0.082	0.002	2.1	0.003	5428	3710	1.46
Pt	L1	0.043	0.001	2.5	0.001	2409	549	1.03

Table S25. TXRF data for the measured single crystal of **6**.

Element	Line	Conc./ umr	Sigma/ umr	RSD/ %	LLD/ umr	Net area	Backgr.	Chi
Si	K12	1240.4	5.5	0.4	1.9	57044	861	5.48
S	K12	10.87	0.34	3.2	0.49	1836	755	1.11
Cl	K12	1.53	0.16	10.3	0.30	426	750	0.90
Ca	K12	0.491	0.053	10.8	0.098	361	573	0.90
Fe	K12	0.373	0.016	4.3	0.025	1270	828	2.26
Ni (IS)	K12	58.69	0.12	0.2	0.02	291484	781	2.93
Cu	K12	0.184	0.009	4.6	0.014	1072	680	1.45
Zn	K12	0.073	0.006	7.8	0.010	496	494	1.57
Br	K12	0.032	0.003	8.7	0.005	342	275	1.04

Table S26. TXRF data for the measured single crystal of **7**.

Element	Line	Conc./ umr	Sigma/ umr	RSD/ %	LLD/ umr	Net area	Backgr.	Chi
Si	K12	72.52	0.38	0.5	0.39	47846	7514	6.41
Cl	K12	64.71	0.13	0.2	0.06	258060	7322	27.31
Mn	K12	0.281	0.005	1.8	0.009	10956	14574	0.88
Fe (IS)	K12	55.850	0.036	0.1	0.005	2731398	5409	95.06
Ni	K12	31.105	0.022	0.1	0.004	2216427	11119	103.85
Zn	K12	0.104	0.001	1.2	0.002	10116	2744	1.29
Br	K12	0.018	0.001	2.8	0.001	2791	1758	4.75

Table S27. Ni/Fe ratio correlation from SCXRD models and TXRF data for compounds **1-7.**

**Compound**	**SCXRD model**		**TXRF data**	
	**Ni**	**Fe**	**Ni**	**Fe**
**1**	1.00	0.00	0.99	0.01
**2A**	0.33	0.66	0.33	0.66
**2B**	0.33	0.66	-	
**3**	0.66	0.33	0.62	0.37
**4**	1.00	0.00	0.88	0.12
**5**	1.00	0.00	1.00	0.00
**6**	1.00	0.00	0.99	0.01
**7**	0.35	0.65	0.36	0.64
